# Public Health Nurses’ Perceptions of Their Roles and Activities Throughout the Phases of the Fukushima Nuclear Disaster: A Qualitative Study

**DOI:** 10.3390/nursrep14040256

**Published:** 2024-11-15

**Authors:** Tamami Koyama, Takumi Yamaguchi, Yuko Matsunari

**Affiliations:** 1 School of Nursing, Tsuruga Nursing University, Fukui 914-0814, Japan; t-koyama@tsuruga-nu.ac.jp; 2 School of Nursing, Tokyo Medical University, Tokyo 160-0022, Japan; 3 Nuclear Safety Research Association, Tokyo 105-0004, Japan; 4 School of Health Sciences, Kagoshima University, Kagoshima 890-0075, Japan; matsuy@health.nop.kagoshima-u.ac.jp; 5 Research Administration Center, Saitama Medical University, Saitama 350-0495, Japan

**Keywords:** Fukushima nuclear disaster, public health nurses, disaster preparedness, qualitative research, radiation emergency, public health nursing activity

## Abstract

**Background/Objectives**: To explore how Public Health Nurses (PHNs) in Fukushima perceived their roles and activities as necessary or inadequate from the immediate aftermath through the long-term recovery of the nuclear disaster. **Methods**: We conducted a qualitative study using a self-administered questionnaire with open-ended questions to capture the perceptions of PHNs across three disaster phases: peacetime; nuclear emergency; and recovery. Responses were analyzed through qualitative content analysis. **Results**: PHNs’ needs and perceived inadequacies varied across the disaster phases. In peacetime, the emphasis was on education for disaster preparedness for both nurses and residents. During the nuclear emergency, the focus shifted to the need for PHN deployment and radiation screening systems, highlighting a significant gap in radiation knowledge. In the recovery phase, the importance of ongoing resident support, rumor management, and trust-building was emphasized, alongside an increased need for radiation education. **Conclusions**: This study highlights the critical need for phase-specific support systems and educational programs to enhance PHNs’ disaster response capabilities. It underscores the importance of preparedness plans and continuous training to improve PHNs’ effectiveness in addressing public health challenges during nuclear disasters. This study was not registered.

## 1. Introduction

The magnitude-9.0 Great East Japan Earthquake struck off the coast of Tohoku, Japan, on 11 March 2011, triggering a massive tsunami that caused widespread destruction and loss of life [1]. The earthquake and tsunami also led to the Fukushima Daiichi Nuclear Power Station accident, which released radioactive materials into the environment [2]. The presence of these materials raised concerns about potential health risks among the affected population. However, a United Nations Scientific Committee on the Effects of Atomic Radiation report indicated that the radiation doses to the public were generally low, and no discernible increase in cancer incidence or other radiation-related diseases was expected [2]. The evacuation of residents from the contaminated areas also led to various health-related problems, such as mental health issues and medical care disruptions [3]. The Fukushima nuclear disaster was the largest nuclear incident since the 1986 Chernobyl accident and has had substantial public health consequences [4].

In the immediate aftermath of the Fukushima nuclear accident, public health nurses (PHNs) in Fukushima Prefecture played a crucial role in responding to the public health needs of the affected population [5]. PHNs were involved in activities including conducting health surveys, providing health consultations, offering mental health support, and communicating risk to the public [6]. These activities included assessing evacuees’ health status, providing medical care, and addressing the specific needs of vulnerable populations, such as older people and pregnant women. However, the disaster’s unprecedented nature posed substantial challenges for PHNs, such as their lack of knowledge about radiation health effects and the difficulty in communicating risks [7].

Several studies have reported on public health nursing activities during specific phases of the Fukushima nuclear disaster [8,9], but there is a lack of comprehensive understanding of the changes in public health nursing roles and activities throughout the disaster timeline. Moreover, the differences in the content of activities and the challenges PHNs face in each disaster phase have not been fully explored.

Investigating the roles and activities that PHNs perceive as necessary or inadequate throughout the Fukushima nuclear disaster’s different phases can provide valuable insights for future nuclear disaster preparedness and response. Accordingly, this study sought to identify the challenges and needs PHNs experienced in each phase, yielding insights that can inform and improve public health nursing preparedness and response in the event of future nuclear disasters. This study, therefore, aimed to qualitatively describe the roles and activities that PHNs during the Fukushima nuclear disaster perceived as necessary or inadequate, from the immediate aftermath to the long-term recovery phase. Through this approach, we sought to answer the central research question, “What roles and activities did PHNs perceive as necessary, and where did they feel inadequacies during each phase of the Fukushima nuclear disaster?”

## 2. Materials and Methods

### 2.1. Study Design and Setting

This cross-sectional study used a self-administered questionnaire targeting PHNs working at Fukushima prefectural offices and health centers in municipalities with populations of at least 250,000. This study was conducted in October 2018, during which questionnaires were distributed to participants, and responses were collected. 

### 2.2. Participants

We distributed a self-administered, anonymous questionnaire by postal mail. A letter requesting participation, a research plan, a questionnaire, and a return envelope were sent to the directors of prefectural and core city health centers in Fukushima Prefecture. The directors were asked to distribute the questionnaire to all PHNs working at their respective health centers. Written consent to participate in this study was implied by the return of the completed questionnaire using the provided self-addressed envelope. 

In total, 68 of 169 nurses (40.2%) responded, and all were included in this analysis.

### 2.3. Data Collection

The questionnaire contained open-ended questions covering what the responding PHNs recognized as necessary and what areas they felt inadequate as PHNs throughout the following nuclear disaster phases [10]:

- Peacetime (under planned exposure situations): February 2011 and earlier;
- Nuclear emergency (under emergency exposure situations): March–December 2011;
- Nuclear disaster recovery (under existing exposure situations): January 2012 onward.

### 2.4. Data Analysis

The descriptive data underwent qualitative content analysis, as Graneheim and Lundman [11] and Elo and Kyngäs [12] described. This analysis included the following steps:

1. Familiarization with the data: Open-ended question responses were read several times to obtain overall comprehension of the contents;
2. Identifying meaning units: The text was divided into condensed meaning units labeled with codes;
3. Coding: The codes were compared for differences and similarities and sorted into categories and sub-categories;
4. Defining and naming categories: The formulated categories were reviewed and refined to ensure clarity and consistency with the coded data;
5. Reporting: The findings were reported as categories and sub-categories, with representative quotes from the original text included to enhance trustworthiness.

In this study, categories were generated for items covering need and the sense of inadequacy, respectively, and the corresponding sense of inadequacy categories were matched to the needed items. We conducted coding and categorizing processes to increase the reliability and validity of the analysis and engaged in discussions to reach a consensus. The authors followed the principles of trustworthiness—including credibility, dependability, and transferability—that Graneheim and Lundman [11] described.

The original data were collected and analyzed in Japanese. The findings were translated into English for reporting purposes after content analysis was completed. We carefully reviewed the translations to ensure that the original meaning was preserved. In the content analysis, we made a summary regarding the sense of inadequacy and the necessary health worker activities described by PHNs for each nuclear disaster phase. Core categories were then generated based on the common content of roles and activities.

## 3. Results

### 3.1. Characteristics of Study Participants

Table 1 shows this study’s participants’ characteristics. The largest proportion (44.1%) held the position of attendant, and approximately 70% worked outside the urgent protective action planning zone.

### 3.2. Recognition of PHNs in Each Disaster Phase

**Figure 1:**
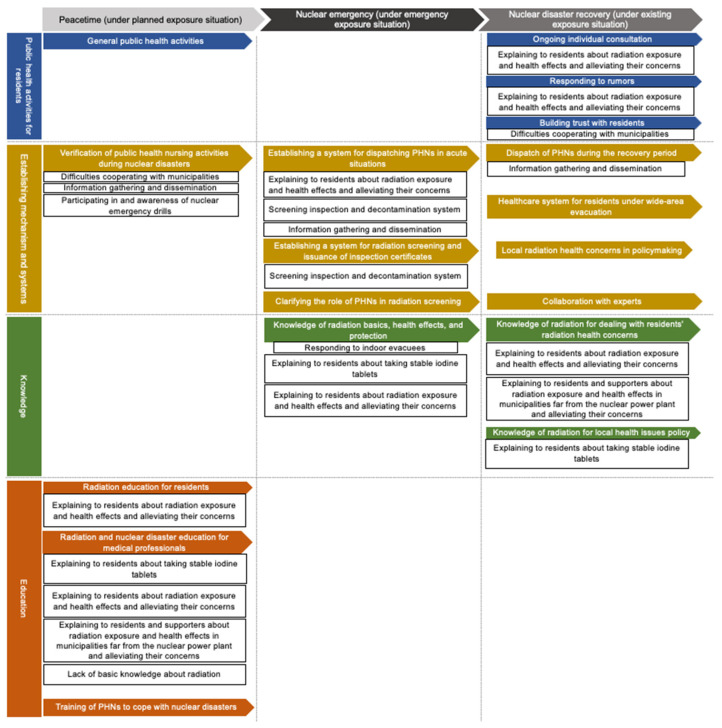
PHNs’ Perceptions of Their Necessities and Inadequacy of Activities throughout the Phases of the Fukushima Nuclear Disaster.

Figure 1 shows the PHNs’ recognition in each disaster phase. The vertical text shows the core categories (public health activities for residents, establishing mechanisms and systems, knowledge, and education). The horizontal axis shows the phases (peacetime, nuclear emergency, and nuclear disaster recovery).

Colored arrows represent the PHNs’ needs during the medium-to-long-term periods from the disaster onset. The related sense of inadequacy is indicated under each need. Four core categories were generated based on the contents of each need: public health nurse activities for residents; establishing mechanisms and systems related to nuclear disasters; knowledge; and education. 

Five categories were generated for the peacetime phase: “General public health activities” (category: public health activity for residents); “Verification of public health nursing activities during nuclear disasters” (establishing mechanism and systems); “Radiation education for residents” (education); “Radiation and nuclear disaster education for medical professionals” (education); and “Training of PHNs to cope with nuclear disasters” (education). As shown in the figure, no need categories were located in the core category of “Knowledge”. Additionally, no insufficiency categories were located in “General public health activities” (public health activities for residents) and “Training of PHNs to cope with nuclear disasters” (education). For example, PHNs expressed a strong need for radiation preparedness, particularly through targeted education. One PHN noted, “The current situation reflects the challenges of addressing nuclear accidents that were once thought unlikely” and, “I felt we should have received training or studied radiation earlier”, highlighting the importance of baseline knowledge in nuclear safety, even during non-emergency periods.

Four categories were generated for the nuclear emergency phase: “Establishing a system for dispatching PHNs in acute situations” (establishing mechanism and systems); “Establishing a system for radiation screening and issuance of inspection certificates” (establishing mechanism and systems); “Clarifying the role of PHNs in radiation screening” (establishing mechanism and systems); and “Knowledge of radiation basics, health effects, and protection” (knowledge). No need categories were located in “Public health activities for residents” and “Education”. No insufficiency categories were located in “Clarifying the role of PHNs in radiation screening” (establishing mechanisms and systems). During the emergency, PHNs encountered significant challenges in conducting radiation screenings. One PHN shared, “At the beginning of the nuclear incident, health centers were overwhelmed with evacuated residents seeking screening tests, which did not fully address their concerns about radiation health effects”. This illustrates the knowledge gaps that complicated PHNs’ efforts to respond effectively in high-pressure situations.

Nine categories were generated for the nuclear disaster recovery phase: “Ongoing individual consultation” (public health activities for residents); “Responding to rumors” (public health activities for residents); “Building trust with residents” (public health activities for residents); “Dispatch of PHNs during the recovery period” (establishing mechanism and systems); “Healthcare system for residents under wide-area evacuation” (establishing mechanism and systems); “Local radiation health concerns in policymaking” (establishing mechanism and systems); “Collaboration with experts” (establishing mechanism and systems); “Knowledge of radiation for dealing with residents’ radiation health concerns” (knowledge); and “Knowledge of radiation for local health issues policy” (knowledge). No need categories were located in “Education”. No insufficiency categories were located in “Healthcare system for residents under wide-area evacuation” (establishing mechanism and systems), “Local radiation health concerns in policymaking” (establishing mechanism and systems), and “Collaboration with experts” (establishing mechanism and systems). In the long-term recovery phase, PHNs noted the need for ongoing mental health support for residents. As one PHN described, “We were uncertain about how to guide outdoor activities in areas previously designated for indoor evacuation; while consultations were needed, conducting visits proved challenging”. This underscores the evolving role of PHNs in addressing both physical and psychological health concerns within the community.

## 4. Discussion

This study qualitatively analyzed PHNs’ perceptions over the medium-to-long term after the Fukushima Daiichi Nuclear Power Station accident, dividing the perceptions into three phases: peacetime; nuclear emergency; and nuclear disaster recovery. The findings revealed that the activities PHNs recognized as necessary and the areas in which they felt inadequate varied across each phase.

- **Peacetime Phase**: PHNs highlighted the importance of preparedness through radiation education for both healthcare professionals and residents, along with regular disaster response training to enhance readiness for potential nuclear incidents. The need for general knowledge was less emphasized in this phase;
- **Nuclear Emergency Phase**: During the emergency phase, PHNs identified the need for an efficient system for PHN deployment and radiation screening. However, many PHNs felt insufficiently prepared due to a lack of knowledge about radiation health effects and protection measures;
- **Recovery Phase**: In the recovery phase, PHNs underscored the need for continuous support, including addressing misinformation, building trust with residents, and providing ongoing health consultations. This phase also highlighted the increased need for specialized knowledge in radiation health management.

These findings underscore the dynamic roles of PHNs across disaster phases, indicating the importance of phase-specific support systems, radiation knowledge, and continuous training to strengthen public health nursing responses to nuclear disasters. Throughout the period, four core categories emerged: public health nursing activities for residents; establishing mechanisms and systems related to nuclear disasters; knowledge; and education.

PHNs are professionals who use the skills acquired in their national certification as PHNs to achieve public health nursing objectives [13] and manage citizens’ health. In health crises, such as any form of disaster, PHNs support public health on the front lines. Outside of times of health crisis management, PHNs, as a matter of course, recognize the need to practice general public health nursing as professionals managing the population’s health from the perspective of preventive medicine. However, though the PHN role is expected during a nuclear disaster emergency [14,15], it has not been recognized as an essential role. PHNs, in reports after the Great East Japan Earthquake, reported their lack of knowledge about radiation, embarrassment at not being able to fully comprehend the process of taking stable iodine tablets, and regret about not being able to provide first aid amid orders to seek shelter indoors [16].

In the core category “Establishing mechanisms and systems”, we believed that the activities required in any disaster phase were recognized and the need for system development was acknowledged. A prefectural emergency medical exposure manual existed at the time of the Fukushima nuclear accident, but it was not customary to read it carefully during times of peace. The nuclear accident was unexpected in the regional disaster prevention plan and public health nursing activity manual, and PHNs were afraid of radiation exposure and resulting health effects and the system for radiation screening tests and issuing inspection certificates [17]. Considering the sense of inadequacy in practicing public health nursing in the event of a nuclear disaster while facing the difficulty of dealing with distressed residents, the PHNs evidently recognized the need to establish a system and structure related to nuclear disasters regardless of the disaster phase.

In the core category of “Knowledge”, knowledge was perceived as necessary during the nuclear emergency and recovery period, while at the same time, PHNs felt inadequate in this area. The PHNs reported struggling in their activities supporting residents during the disaster because they were unable to judge what information was correct and what information to convey to residents in the absence of basic knowledge and information on radiation [18]. This study also found recognition that knowledge about radiation was necessary during nuclear disaster emergencies and recovery. In addition to the perceived inadequacies in knowledge among PHNs in Fukushima, international perspectives on radiation risk perception further highlight the influence of cultural and social contexts on public health responses. For example, a study conducted in Serbia demonstrated that residents experience a complex conflict between fear of radiation exposure and the perceived social benefits of nuclear power [19]. This finding suggests that social and cultural backgrounds significantly shape how populations perceive radiation risks, which, in turn, influences how health professionals communicate and support affected communities. In Fukushima, PHNs played a critical role in managing both the physical and psychological health concerns of residents, often facing challenges in building trust and effectively communicating risk information amidst high levels of fear and uncertainty. By comparing these findings with international studies, such as the Serbian case, it becomes clear that the role of PHNs during nuclear disasters must adapt to the social contexts in which they operate. This international comparison underscores the importance of culturally sensitive risk communication strategies and suggests that strengthening PHN knowledge on radiation in diverse contexts could improve disaster response and public health resilience worldwide.

In the core category of “Education”, the PHNs clearly felt the need to undergo education during peacetime. We found that, in the aftermath of the Fukushima accident, they needed knowledge about nuclear disasters and educating residents as part of their own preparedness for nuclear disasters. They were aware of the need for education on radiation and nuclear disasters under normal circumstances. In public health nursing conducted in the medium-to-long-term period following the Fukushima accident, PHNs also received evacuees from a wide area and set up evacuation centers. Additionally, PHNs were dispatched from other prefectures on request to provide health support to residents in the affected municipalities. The PHNs had difficulty dealing with residents who were concerned about radiation’s health effects. PHNs in all municipalities need to be educated during times of peace, regardless of whether there are nuclear facilities in their areas [20].

### Limitations 

This study has several strengths. It provides valuable insights into the perceptions of PHNs across different phases of a nuclear disaster, a topic that previous research has not comprehensively explored. The qualitative approach allowed for a deep understanding of PHNs’ experiences and perspectives. This study included PHNs from various health centers in Fukushima Prefecture, ensuring a diverse range of experiences and opinions. The findings also have important implications for developing phase-specific support systems and educational programs for PHNs about nuclear disaster preparedness and response.

This study also has some limitations. First, the findings’ generalizability may be limited, as this study only focused on PHNs working in health centers in Fukushima Prefecture. This study relied on self-reported data from a questionnaire survey, which had inherent limitations. Additionally, as demographic data such as age, gender, and years of experience were not collected in the questionnaire, it was not possible to analyze how these factors might have influenced PHNs’ perceptions and responses. Future research would benefit from including demographic questions to capture a broader understanding of the characteristics that may shape PHNs’ roles and experiences during nuclear disaster response. As this was a cross-sectional study, it also has limitations in determining causal relationships. Finally, this study concentrates on the perceptions of PHNs and does not include the perspectives of other stakeholders, such as residents, other healthcare professionals, and administrative officials.

## 5. Conclusions

This qualitative study explored the perceptions of PHNs in Fukushima Prefecture, Japan, regarding their roles and activities throughout the phases of the Fukushima nuclear disaster: peacetime, nuclear emergency, and recovery. The findings show that the PHNs’ perceived needs and inadequacies varied substantially across the phases, highlighting the importance of developing phase-specific support systems, educational programs, and disaster preparedness plans. This study underscores the need for ongoing education and training for PHNs in nuclear disaster preparedness, regardless of whether there are nuclear facilities in their areas. Incorporating lessons learned from the Fukushima experience into PHN education and disaster management plans can help build a more resilient public health workforce. Despite the study’s limitations in terms of generalizability and reliance on self-reported data, it provides valuable insights into the PHNs’ complex and evolving roles in a nuclear disaster context. Future research should include a broader range of participants, explore other stakeholders’ perspectives, and employ longitudinal designs to better understand nuclear disasters’ long-term impacts on public health nursing. This study emphasizes the need for phase-specific support, education, and preparedness to strengthen the capacity of PHNs to effectively respond to nuclear disasters. Addressing the identified challenges and leveraging the lessons learned from the Fukushima experience can help safeguard the health of affected communities in future nuclear disasters.

## Figures and Tables

**Table 1:** Characteristics of study participants (*n* = 68).

Item	Response	n	%
**Position**	No response	4	5.9
	Reappointment	2	2.9
	Attendant	30	44.1
	Unit chief	20	29.4
	Assistant manager	2	2.9
	Manager	6	8.8
	Deputy director	3	4.4
	Director	1	1.5
**Working place**	Within UPZ	17	25.0
	Outside UPZ	47	69.1
	No response	4	5.9

## Data Availability

All data are available from the corresponding author upon reasonable request.

## Public Involvement Statement

No public involvement in any aspect of this research.

## Guidelines and Standards Statement

This manuscript was drafted against Standards for Reporting Qualitative Research (SRQR).
